# Machine Learning From Molecular Dynamics Trajectories to Predict Caspase-8 Inhibitors Against Alzheimer’s Disease

**DOI:** 10.3389/fphar.2019.00780

**Published:** 2019-07-12

**Authors:** Salma Jamal, Abhinav Grover, Sonam Grover

**Affiliations:** ^1^JH-Institute of Molecular Medicine, Jamia Hamdard, New Delhi, India; ^2^School of Biotechnology, Jawaharlal Nehru University, New Delhi, India

**Keywords:** Alzheimer’s, caspase-8, machine learning, molecular dynamics trajectories, descriptors

## Abstract

Alzheimer’s disease (AD) is a neurodegenerative disorder in which the death of brain cells takes place leading to loss of memory and decreased cognitive ability. AD is a leading cause of death worldwide and is progressive in nature with symptoms worsening over time. Machine learning–based computational predictive models based on 2D and 3D descriptors have been effective in identifying potential active compounds. However, the use of data from molecular dynamics (MD) trajectories for training machine learning models still needs to be explored. In the present study, descriptors have been extracted from the MD trajectories of caspase-8 ligand complexes to train models using artificial neural networks and random forest algorithms. Caspase-8 plays a key role in causing AD by cleaving amyloid precursor proteins during apoptosis leading to increased formation of the amyloid-beta peptide. A total of 43 ligands were docked using the glide module of Schrodinger software, and short MD simulations of 10 ns were performed for the calculation of MD descriptors. The MD descriptors were also combined with the 2D and 3D descriptors of chemical compounds, and individual descriptor based as well as combination models were generated. This study demonstrated that MD descriptors could be effectively used for the characterization of bioactive compounds along with lead prioritization and optimization.

## Introduction

Neurological disorders affect millions of people globally with Alzheimer’s disease being the most common type of disease. Alzheimer’s disease (AD) is the sixth prominent cause of death in the United States and, as per the data from the National Center for Health Statistics of the center for disease control (CDC), AD was responsible for approximately 110,561 deaths in 2015 (Alzheimer’s Association, 2018). AD is the only disease among the top 10 causes of death with no means of prevention, treatment, or delay in progression. The disease is pathologically defined by protein aggregation and its impact on the function of neurons; therefore, studies have been primarily focusing on reducing protein aggregation and promoting clearance from the brain (Small et al., 2001). However, these therapies have been unsuccessful in clinical trials, which suggests targeting protein aggregation and clearance alone may not be sufficient to treat AD. Among the many factors responsible for AD such as amyloid hypothesis, cholinergic hypothesis, tau hypothesis, environmental risks, and genetic factors, it has been well established that approximately 70% of risk for the disease is attributable to genetics (Ballard et al., 2011). The previously discovered genes presenilin 1 (*PSEN1*), presenilin 1 (*PSEN2*), and amyloid precursor protein (*APP*) are accountable for the pathogenesis of AD in only about 5% of patients (Van Cauwenberghe et al., 2016). Considering the complex physiology of AD and multiple causes responsible for the disease, drug development against AD must consider all events related to the pathophysiology for more effective treatment strategies, which cannot be accomplished concentrating on one cause alone. The development of effective treatment options for AD has been of great interest considering the global burden of the disease, and thus, identification of more potent and selective inhibitors from the large pool of chemical compounds is imperative. Caspases have been reported to play an important role in AD due to the increase in β-amyloid levels by the cleavage of APP during apoptosis (Rohn et al., 2001). Multiple evidences are there which suggest that APP is a substrate for caspase-lead cleavage which is a crucial step in the AD process that may result in amyloid-beta formation, loss of synaptic activity, and behavior changes related with AD (Gervais et al., 1999; Cotman et al., 2005; Galvan et al., 2006). Recent studies have reported that activation of caspases leads to the formation of neurofibrillary tangles (NFT) (Gamblin et al., 2003; Rissman et al., 2004). Another study has confirmed the cleavage of tau by caspases in the early state of AD (Guillozet-Bongaarts et al., 2005). It has also been put forward that caspase-mediated truncation of tau is interrelated with the development of NFTs and beta-amyloids in AD (Dickson, 2004). In addition, all the caspases -1, -2, -3, -5, -6, -7, -8, and -9 have been identified to be transcriptionally elevated in AD (Castro et al., 2010). Caspase-8 has been labeled an originator caspase that further activates other downstream caspases, making this enzyme an attractive target for the identification and development of inhibitors. This could prevent unwanted cell death related to various neurodegenerative disorders (Watt et al., 1999). Caspase-8 has also been associated with synaptic plasticity as well as associated neurotoxicity through its downstream effector caspase-3, which points toward other supplementary mechanisms that might lead to AD (Rehker et al., 2017). Caspases play an important role in disease mechanisms associated with AD that include formation of beta-amyloids as well as NFTs and thus inhibiting caspases may lead to prevention of formation of plaques and tangles and also reducing disease progression. Computational predictive models have been of great use to researchers doing studies on drug discovery. Machine learning approaches have been used extensively for the identification of potential active compounds based on 2D and 3D molecular descriptors (Jamal et al., 2015; Wahi et al., 2015; Jamal et al., 2017). Although the previously developed models were successful for screening lakhs to millions of compounds, a high degree of reliability is required for prioritizing the top five or 10 compounds from a set of hundreds of possibilities. This necessitates the generation of more accurate hyper-predictive target-specific models utilizing the descriptors extracted from molecular dynamics (MD) trajectories and consideration of protein-ligand interactions (Ash and Fourches, 2017). Various quantitative structure activity relationship studies for the development of caspase-3 inhibitors have already been reported in the literature (Legewie et al., 2006; Wang et al., 2009; Firoozpour et al., 2012; Sharma et al., 2013). The present study was carried out to utilize the potential of MD-derived descriptors in predictive modeling of potent caspase inhibitors. Thus, the present study is based on the hypothesis that MD-based machine learning models could be extremely useful for lead optimization and chemical compound prioritization. Potential inhibitors of caspase-8 have been used for the calculation of 2D, 3D, and MD descriptors. The ligands were docked into caspase-8 protein, and the protein-ligand complexes were subjected to MD simulations to generate descriptors from MD trajectories. Further, artificial neural network and random forest machine learning algorithms were used to generate the models using an individual set of descriptors with two and three level combinations. The conformational dynamics of caspase-8 upon binding with the compound predicted to be active against the protein using 100 ns MD simulation was also explored. Moreover, pharmacophore model was developed using the ligands associated with caspase-8 which was further used for virtual screening to identify the new potential caspase inhibitors.

## Methodology

### Caspase-8 Data Set

In the present study, we used the caspase-8 data set comprised of ligands associated with caspase-8 retrieved from the ChEMBL (Gaulton et al., 2012) database (ChEMBL46860, ChEMBL46862, ChEMBL399983, ChEMBL304686, ChEMBL430105, ChEMBL46849, and ChEMBL741342). A total of 81 compounds were obtained and preprocessed (Fourches et al., 2010), during which duplicates and compounds with approximate IC50 values were removed. Post-processing the data resulted in 43 compounds with pIC50 values ranging from 4.3 to 8.1, among which compounds with a pIC50 value above 6.5, were considered as active compounds while those with a pIC50 below 6.5 were considered as inactive compounds. The final data set including the molecule identifiers, SMILES, and pIC50 values has been provided in the Supporting Information. 

### Molecular Docking

The X-ray crystal structure of human caspase-8 (PDB ID: 1qtn) in complex with acetyl-ile-glu-thr-asp-aldehyde peptide at a resolution of 1.2 Å was obtained from the protein data bank (PDB) (Parasuraman, 2012). The protein-ligand complex was preprocessed using Accelrys ViewerLite (Accelrys Inc., San Diego, CA, USA) during which ligands, water molecules, and heteroatoms were removed. Further, the protein was prepared with Preparation Wizard available from Schrodinger Suite (http://www.schrodinger.com/). Hydrogen bonds were added, and bond orders were assigned during protein preparation. The protonation states of residues were predicted using the PROPKA (Olsson et al., 2011) program at pH 7 followed by minimization of the protein using the OPLS3 force field (Sastry et al., 2013). The ligands associated with the caspase-8 protein were prepared using the LigPrep (Schrödinger, Inc., www.schrodinger.com) module of Schrodinger before molecular docking. The ligands were also minimized using the OPLS3 force field, and the possible ionization states were created at pH 7.0 ± 2.0. The tautomers were generated, specific chiralities of the ligands were retained, and 32 conformations per ligand were generated in case of indefinite chiralities. Next, using the Receptor Grid Generation section of the Glide (Halgren et al., 2004) module of Schrodinger, the binding site in protein was defined using the centroid of selected residues option in which the catalytic triad Cys360, His317, and Arg258 were chosen. A scaling factor of 1.0 was used to scale the van der Waals radii of receptor atoms having a partial atomic charge less than the specified cut-off, which was equal to 0.25. All other parameters were default. The prepared ligands were then docked into the active site of the receptor using an extra precision algorithm of Glide. The top-ranked pose for each ligand was selected and subjected to MD simulation studies.

### Molecular Dynamics Simulation Details

The top scoring protein-ligand complexes were subjected to 10-ns MD simulations to evaluate their structural and thermodynamic stability in the presence of explicit salt and solvents. All the MD simulation studies were performed using the GROMACS (Abraham et al., 2015) software version 5.0 and GROMOS96 force field. Prior to the MD simulation, each protein-ligand complex was prepared by the removal of the water molecules, addition of hydrogen atoms, capping of termini, treating disulphides, and finding overlaps. After the initial preparation, the model was solvated with a simple point charge (SPC) water model and Na+ and Cl- ions were added to maintain the neutrality of the system. The solvated system was then subjected to energy minimization for 50,000 steps using the steepest descent method until a maximum force of 10.0 kJ/mol was attained. An equilibration run was performed in two sequential steps, NVT (number of particles, volume, and temperature) equilibration, and NPT (number of particles, pressure, and temperature) equilibration during which pressure and temperature were kept to 1 bar and 300°C, respectively, for a maximum of 50,000 steps in both the types of equilibration. Further, a 10-ns MD simulation run was carried out to obtain a stable structure and time *versus* RMSD (root-mean square deviation) plot to ensure the stability of the system for the entire simulation run. 

### Descriptors Computation

Molecular descriptors represent the chemical information of the ligands using numeric values. Three types of descriptors were used for modeling in the present study, 2D, 3D, and MD descriptors. The 2D descriptors included atom count, bond count, carbon types, hydrogen bond donor and acceptor count, Lipinski’s rule of five, rotatable bonds count, topological surface area, van der Waals volume, and many more. The 3D descriptors included gravitational index descriptor, charged partial surface area, and length over breadth and moment of inertia descriptors, among others. The 3D-WHIM descriptors involved descriptors weighted by unit weights, van der Waals volumes, atomic masses, atomic polarizabilities, and Mulliken atomic electronegativites. A total of 770 2D descriptors and 115 3D descriptors were generated for each ligand conformation using PaDEL (Yap, 2011) software. 

For MD descriptors, the trajectory of each protein-ligand complex was analyzed for three properties, radius of gyration (Rg), potential energy and total energy, and solvent accessible surface area (SASA). Each of the three MD descriptors was represented using the mean and standard deviation as described in other studies (Ash and Fourches, 2017; Riniker, 2017), resulting in a total of eight descriptors. 

### Model Building 

Machine learning (ML)–based modeling is learning from known properties and using the learned model systems to make predictions for unseen data. Using an in-house Perl script, the molecular descriptor files were split with 70% for a training set and 30% for a testing data set. The training set was used for generation of the models, and the test set was used for the assessment of model performance. An internal validation of the models generated using the training set was performed using *k*-fold cross validation, with *k* equal to 10 in the present work. Cross validation is a technique in which the data is divided into *k* subsets, with *k*-1 subsets used for model generation and the remaining subset used for testing purposes. This process is repeated until all the *k* folds have been used as a testing set at least once. The models were generated using individual 2D, 3D, and MD descriptors and their two level 2D+3D, 2D+MD, and 3D+MD and three level 2D+3D+MD combinations. The 2D, 3D, MD, 2D+3D, 2D+MD, 3D+MD, and 2D+3D+MD artificial neural network (ANN) and random forest (RF) models were generated using different parameters and finding the best combination of parameters. 

### Machine Learning Algorithms

In the present study, two ML algorithms, ANN and RF, were used for building the models using Weka which is an ML software. ANN is a computational model that attempts to mimic the structure and function of neural networks in the human brain. It comprises a group of connected artificial neurons that process information and generate output. The ANN model used in the present study is multilayer perceptron (MLP), which is a feedforward ANN model using three or more layers including input and output layers along with hidden layers, to map input data and produce the correct output (Cheng et al., 2008). 

RF is a decision tree based classifier that creates an assembly of decision trees and outputs the class that is the mode of the output of all the individual decision trees. The decision tree for each attribute is created by sampling the attributes, then using random selection. Next, the information gain criterion is used to select the best feature from the data which is used as the origin node of the tree. The origin node is then divided into sub-nodes, and the process is repeated until the sub-node becomes an output class. The final prediction is the class chosen by the majority of the trees (Breiman, 2001).

### Feature Selection

Feature, attribute, or descriptor selection is the procedure of identifying a subgroup of features that are relevant to the modeling and prediction task. Feature selection is performed to decrease the dimensionality of the data by eliminating insignificant features and thus reducing training time, removing redundant descriptors, simplifying models, and lessening overfitting of the models. Feature selection was performed at two levels, initially using the Remove Useless filter of the Weka (Bouckaert et al., 2010) ML tool followed by the selection of significant features. The Weka Remove Useless filter removed the descriptors having the same value for all compounds, as those descriptors did not contribute toward classification.

Two feature selection techniques were used, correlation-based feature selection (CFS) and relief attribute evaluation. CFS ranks features using a correlation based heuristic function which outputs a subset of features having a high correlation with the class but uncorrelated with each other (Hall, 1999). The following correlation based heuristic function is used for calculating the merits of a feature subset:

$$Ms=\frac{k\bar{rcf}}{k+k(k-1)\bar{rff}}$$

where *Ms* is the merit of feature subset *S* consisting of *k* features, *rff* is the mean of feature-feature correlation, and *rcf* is the mean of feature-class correlation. 

The relief-based attribute selection algorithm calculates a feature score, ranks the features, and chooses the top ranked. The feature score is calculated using the Euclidean distances between features and their nearest neighboring instances. The training data set was used for feature selection, and the test set used to rid the data of any biasness (Kira and Rendell, 1992).

### Model Performance Evaluation

A total of 14 ML models were generated using ANN and RF algorithms, which were evaluated using accuracy, balanced accuracy, training error, generalization error, and a receiver-operating characteristic (ROC) plot. Accuracy ([{TP+TN/(TP+TN+FP+FN}]) is the proportion of correctly classified active and inactive compounds by the classification models. An ROC plot is a graph plotted as true positive rate (TPR) *vs* false positive rate (FPR, 1-specificity). TPR ([TP/{TP+FN}]) is the percentage of correctly classified actives while FPR (1-[TN/{TN+FP}]) is the proportion of correctly identified negatives.

### Pharmacophore Search and Virtual Screening 

The 43 compounds used for the generation of ML models were used for ligand-based pharmacophore modeling using PharmaGist tool (Schneidman-Duhovny et al., 2008). A pharmacophore is a theoretical representation of features of ligand necessary for the recognition of ligand by the macromolecule and can be used to identify ligands that can bind to a common receptor through virtual screening. PharmaGist tools search for probable pharmacophores by multiple flexible alignment of input ligands and report the top scoring ones. The pharmacophore model developed in the present study was used for virtual screening to search through a total of 1,798 and 16 natural compounds from ZINCPharmer (Koes and Camacho, 2012) to get the similar hits from ZINC database. The top 10 most similar hits were subjected to Glide’s XP docking with the caspase-8 protein used for the docking study with 43 ligands used in the present study.

## Results 

### Glide-Docking Analysis

**Figure 1:**
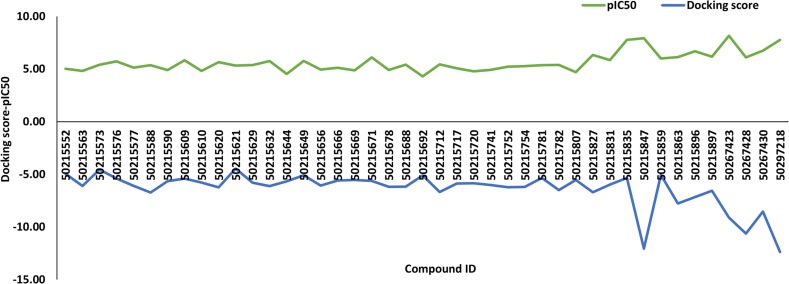
The correlation plot between docking scores and pIC50.

A total of 43 active and inactive caspase-8-associated ligands were docked in the active site of the receptor protein, human caspase-8, using the extra precision (XP) docking approach. The XP docking scores of ligands ranged from −12.70 to −4.22 kcal/mol. The compounds having a pIC50 value above 6.5 categorized as actives corresponding to compound IDs 50267423, 50215849, 50215847, 50215835, 50297218, 50267430, and 50215896 had docking scores of −9.1 kcal/mol, −6.22 kcal/mol, −12.06 kcal/mol, −5.34 kcal/mol, −12.38 kcal/mol, −8.53 kcal/mol, and −7.16 kcal/mol, respectively. Additionally, we have generated the correlation plot between docking scores and pIC50. As is evident from the plot, the compounds having high pIC50 values had higher docking scores and *vice versa* (**Figure 1**).

### Feature Analysis

In the present study, feature analysis was performed using the Remove Useless filter, CFS, and relief-based attribute selection using Weka. The number of 2D descriptors was reduced from 770 to 387 after the Remove Useless filter was applied. The numbers of 3D and MD descriptors remained the same, 115 and 8, respectively. 

E-state indices represent the electronic and topological character of an atom where the electronic state of an atom is encoded as perturbed by the electronic impact of other atoms in the molecule in context of the topological character of the molecule. The top ranked 2D features selected using CFS and relief-based selection included nwHBd, SwHBd, SHCHnX, minHCHnX, minwHBd, maxwHBd, maxHCHnX, and nHCHnX. The selected 2D features included count, sum, and minimum and maximum of E-states for weak hydrogen bond (H-bond) donors and atom type, H (estate: CHnX where nX corresponds to a halogen atom). Various studies have explained the importance of weak H-bonds in chemical and biological systems (Steiner, 1999). 

The top ranked 3D features included FPSA-3, WK.unity, Wnu2.unity, WK.mass, Wnu2.mass, Weta3.volume, Wlambda3.mass, and TPSA. The selected 3D descriptors included WHIM descriptors which capture significant molecular 3D information that include shape, molecular size, atom distribution, and symmetry. These indices are computed using *x*, *y*, and *z* coordinates of a molecule using different weighing schemes like atomic mass, van der Waals volume, electronegativity, and atomic polarizabilities and have been used for QSAR modeling (Gramatica, 1997). 

In the case of MD descriptors, all the eight descriptors that included mean and standard deviation of potential and total energy, Rg, and SASA were used for model generation. The MD descriptors included total and potential energy, Rg, and SASA where total and potential energy are mathematical forms of representations of protein-ligand interactions; Rg indicates the compactness of the protein, and that is how the secondary structures are compactly folded in to 3D structure of the protein. SASA is a measure of accessible surface of a molecule which further helps in secondary structure prediction. The number and description of features used in the present study have been provided in **Table 1**.

**Table 1:** The number and description of features used in the present study.

Type of descriptor	Initial number of descripted	Remove useless filter	Relief-based selection	Selected descriptors	Description as provided by PaDEL
2D	770	387	8	nwHBd, SwHBd, SHCHnX, minHCHnX minwHBd, maxwHBd, maxHCHnX nHCHnX	Atom type electrotopological state Count of E-state for weak H-bond donors Sum of E-state for weak H-bond donors Sum of atom type H E-state: CHnX Minimum atom type H E-state: CHnX Minimum of E-state for weak H-bond donors Maximum of E-state for weak H-bond donors Maximum atom type H E-state: CHnX Count of atom type H E-state: CHnX
3D	115	115	8	FPSA-3, WK.unity, Wnu2.unity, WK.mass, Wnu2.mass, Weta3.volume, Wlambda3.mass TPSA	Charged partial surface area Non-directional WHIM weighted by unit weights Directional WHIM weighted by unit weights Non-directional WHIM weighted by atomic masses Directional WHIM weighted by atomic masses Directional WHIM weighted by van der Waals volumes Directional WHIM weighted by atomic masses Topological polar surface area
MD	8	8	8	Potential energy Total energy Radius of gyration Solvent accessible surface area	

In addition to this, we also calculated importance of MD descriptors. This was carried out by computing average merit and average rank using CFS, relief-based attribute selection, and classifier attribute evaluation using ANN and RF classifiers. Average merit indicates the average accuracy loss when a particular feature is removed whereas average rank denotes the rank of the feature determined using 10-fold cross validation. The results indicated that potential energy of the protein-ligand complex was the most significant contributor toward classification followed by Rg, SASA, and total energy (**Table 2**).

**Table 2:** Importance of molecular dynamics (MD) descriptors using correlation-based feature selection (CFS), relief-based attribute selection, and classifier attribute evaluation using artificial neural network (ANN) and random forest (RF) classifiers.

MD descriptor	CFS		Relief-based		Classifier attribute evaluator (ANN)		Classifier attribute evaluator (RF)	
	Average merit	Average rank	Average merit	Average rank	Average merit	Average rank	Average merit	Average rank
Total energy	0.062 ± 0.032	2.6 ± 0.92	0.021 ± 0.028	3.3 ± 0.64	−0.015 ± 0.023	2.2 ± 1.47	−0.133 ± 0.032	3.8 ± 0.4
Potential energy	0.062 ± 0.032	1.8 ± 0.75	0.021 ± 0.028	2.3 ± 0.64	−0.015 ± 0.023	2.4 ± 0.49	−0.068 ± 0.046	2.5 ± 0.81
Gyration	0.048 ± 0.033	2.9 ± 1.14	0.014 ± 0.011	3.2 ± 0.98	0 ± 0	2.6 ± 0.49	−0.036 ± 0.024	1.8 ± 0.75
SASA	0.057 ± 0.041	2.7 ± 1.27	0.092 ± 0.013	1.2 ± 0.6	0 ± 0	2.8 ± 1.47	−0.028 ± 0.036	1.9 ± 1.04

### Model Predictions and Performances

**Figure 2:**
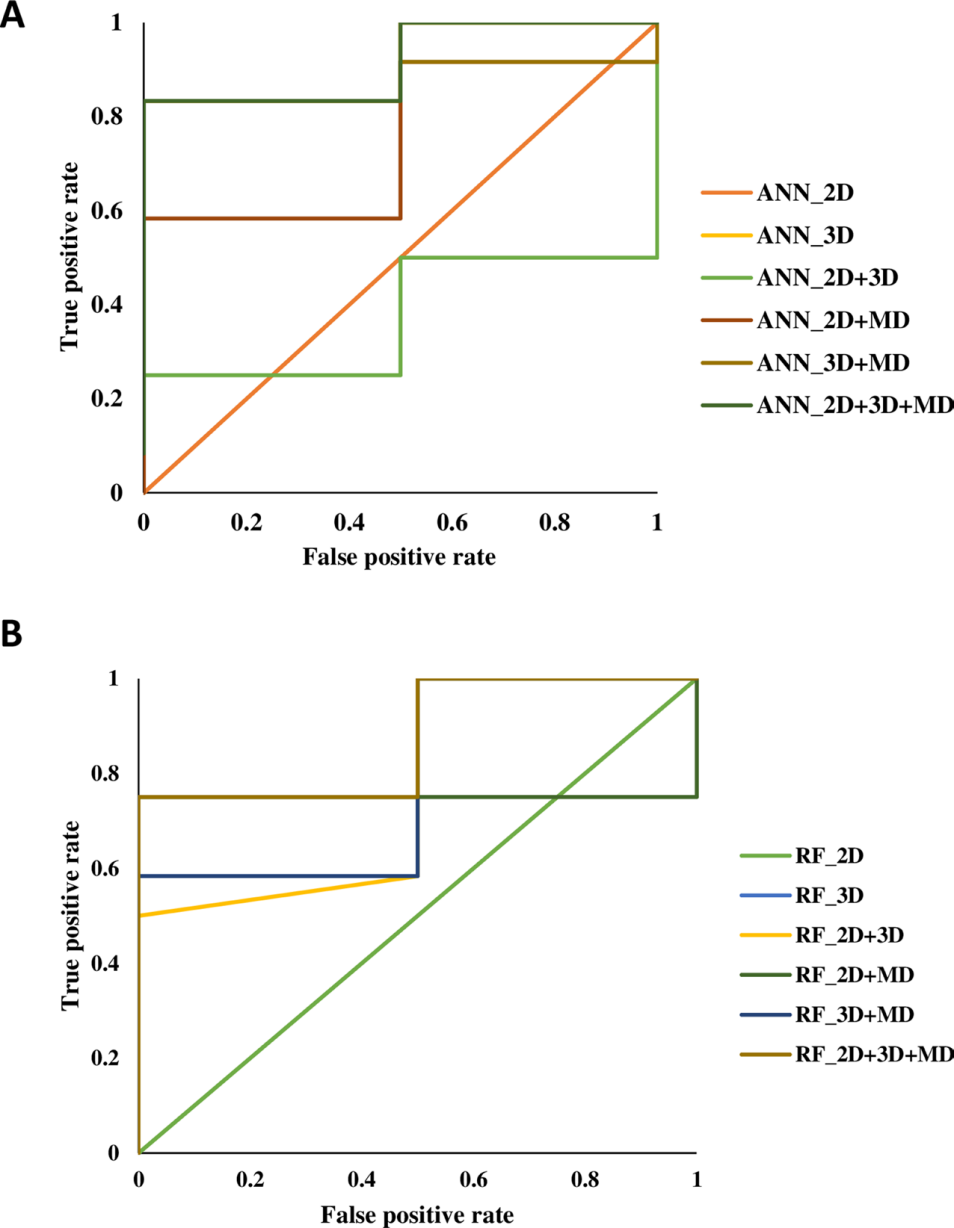
The receiver-operating characteristic (ROC) plots generated for artificial neural network (ANN) and random forest (RF) models using 2D, 3D, MD, 2D+3D, 2D+MD, 3D+MD, and 2D+3D+MD descriptors.

A total of 14 ML models were generated in the present study using ANN and RF ML algorithms. These ANN and RF models (2D, 3D, MD, 2D+3D, 2D+MD, 3D+MD, and 2D+3D+MD) were generated using best combination of different parameters. Initially, we tried to the models using default parameters for ANN and RF algorithms. However, these did not perform well in terms of the statistical parameters used for model performance evaluation (**Table 3**). The training set consisted of 29 compounds, and the test data set consisted of 14 compounds. **Table 4** provides the performance metrics of all the generated ANN and RF models using the best combination of parameters. **Figure 2** illustrates the ROC plots generated for ANN and RF models using 2D, 3D, MD, 2D+3D, 2D+MD, 3D+MD, and 2D+3D+MD descriptors. The training and testing sets used for generating the models and the models build in the present study have been provided as **Supplementary Material**.

**Table 3:** The performance metrics of all the generated machine learning (ML) models using ANN and RF algorithms using default parameters.

Machine learning algorithm	Descriptor type	Cross-validation accuracy (%)	Accuracy (%)	AUC	Balanced accuracy (%)	Training error	Generalization error	Training error	Generalization error
						MSE	RMSE	MSE	RMSE
Artificial neural network	2D	86.20	85.71	0.50	50.00	0.21	0.35	0.20	0.35
	3D	82.75	85.71	0.91	70.50	0.16	0.37	0.16	0.37
	MD	82.75	85.71	0.66	50.00	0.27	0.39	0.22	0.34
	2D+3D	89.65	85.71	0.91	70.50	0.10	0.28	0.16	0.38
	2D+MD	75.86	85.71	0.58	50.00	0.29	0.46	0.20	0.35
	3D+MD	89.65	78.57	0.87	66.50	0.10	0.25	0.20	0.42
	2D+3D+MD	89.65	78.57	0.87	66.50	0.13	0.32	0.20	0.42
Random forest	2D	86.20	85.71	0.50	50.00	0.21	0.35	0.21	0.35
	3D	89.65	85.71	0.50	62.00	0.15	0.26	0.18	0.34
	MD	82.75	85.71	0.68	50.00	0.26	0.38	0.19	0.34
	2D+3D	86.20	85.71	0.52	50.00	0.15	0.27	0.18	0.33
	2D+MD	82.75	85.71	0.62	50.00	0.26	0.40	0.19	0.35
	3D+MD	86.20	85.71	0.89	50.00	0.16	0.26	0.17	0.31
	2D+3D+MD	86.20	85.71	0.87	50.00	0.17	0.28	0.16	0.30

**Table 4:** The performance metrics of all the generated ANN and RF models using the best combination of parameters.

Machine learning algorithm	Descriptor type	Cross-validation accuracy (%)	Accuracy (%)	AUC	Balanced accuracy (%)	Training error	Generalization error	Training error	Generalization error
						MSE	RMSE	MSE	RMSE
Artificial neural network	2D	86.20	85.71	0.50	50.00	0.21	0.25	0.2	0.35
	3D	44.82	64.28	0.91	79.15	0.52	0.38	0.44	0.51
	MD	82.75	85.71	0.70	50.00	0.25	0.4	0.21	0.34
	2D+3D	13.79	85.71	0.37	50.00	0.63	0.64	0.47	0.47
	2D+MD	51.72	85.71	0.75	70.85	0.52	0.59	0.42	0.43
	3D+MD	75.86	78.57	0.83	87.50	0.35	0.47	0.33	0.47
	2D+3D+MD	62.06	78.57	0.91	87.50	0.48	0.55	0.39	0.47
Random forest	2D	86.20	85.71	0.50	50.00	0.47	0.47	0.47	0.47
	3D	82.75	78.57	0.79	66.65	0.24	0.35	0.32	0.37
	MD	55.17	71.42	1.00	83.35	0.44	0.53	0.32	0.38
	2D+3D	65.51	57.14	0.77	54.15	0.37	0.43	0.43	0.47
	2D+MD	86.20	85.71	0.75	50.00	0.21	0.38	0.17	0.35
	3D+MD	89.65	92.85	0.79	75.00	0.17	0.26	0.19	0.31
	2D+3D+MD	72.41	85.71	0.91	91.50	0.38	0.42	0.36	0.39

### Modeling Using 2D Descriptors

The 2D ANN and RF models had an accuracy of 85.71%, balanced accuracy of 50.0%, and an AUC value of 0.50. The AUC value indicated these models were random predictors and thus were not considered for further predictions. 

### Modeling Using 3D Descriptors

The 3D descriptor models had an accuracy, balanced accuracy, and AUC value of 64.28%, 79.15%, and 0.91, respectively, for the ANN model. In case of RF model, the accuracy, balanced accuracy, and AUC values corresponded to 78.57%, 66.65%, and 0.79. These results indicated that 3D compound descriptors play a vital role in the classification of compounds. The ANN model correctly predicted the two active compounds 50267423 and 50215896 and the other inactive compounds predicted as active by ANN had compound IDs 50215590, 50215632, 50215692, 50215782, and 50215859. The RF model gave the correct prediction for only one active compound, 50267423. The other inactive compounds predicted as active included 50215590 and 50215632.

### Modeling Using MD Descriptors 

The models generated using MD descriptors had an accuracy of 85.71% and 71.42%, balanced accuracy of 50.0% and 83.35%, and an AUC value of 0.70 and 1.00 for ANN and RF models, respectively. The MD models had the most balanced accuracies and AUC values compared to the 2D and 3D descriptor models, in which either the accuracy was high and AUC value was low or vice versa. This clearly indicates the descriptors extracted from MD trajectories play a significant role in lead prioritization, resulting in most active compounds. The reduction in generalization error as compared to training error indicated that MD descriptors can perform well on new data. The ANN model correctly predicted the inactive compounds, but misclassified the compounds categorized as active. However, the RF model predicted the active compounds, 50267423 and 50215896, as active. The other compounds predicted as active included compounds corresponding to IDs 50215632, 50215720, 50215782, and 50267428.

### Modeling Using the Two Level Combination of 1D, 2D, and MD Descriptors 

The models were generated by combining 1D, 2D, and 3D descriptors as 2D, 2D+MD, and 3D+MD. The 2D+3D descriptor models had an accuracy of 85.71% and 57.14%, balanced accuracy of 50.0% and 54.15%, and an AUC value of 0.37 and 0.77 for ANN and RF models, respectively. The 2D+3D RF model predicted one active compound, 50267423, accurately. In the case of RF models, the accuracy and balanced accuracy of the models remained the same when 2D descriptors were combined with MD descriptors. Although the accuracy was same (85.71%) in case of ANN models (2D and 3D), there was a significant increase in the balanced accuracy (from 50% to 70.85%) and AUC (from 0.37 to 0.75) upon addition of MD descriptors.

When the 3D descriptors were combined with MD descriptors, an increase in accuracy (from 64.28% to 78.57%) and balanced accuracy (from 79.15% to 87.50%) was observed in case of ANN models; however, there was a slight reduction (from 0.91 to 0.83) in the AUC value. In the case of models generated using the RF algorithm, the accuracy (from 78.57% to 92.85%) and balanced accuracy (from 66.65 to 75%) values improved while AUC (0.79) value remained the same in case of addition of MD+3D descriptors. The results clearly indicate the combination of models resulted in greater accuracy with the 3D+MD combination models being the most informative. As the 3D+MD combination models had the best performance, the compounds predicted as active by these models were corresponding to IDs 50267423, 50215590, and 50215720. 

It was also observed that the models generated using 2D and 3D descriptors in combination with MD descriptors had low mean absolute error (MSE) and root mean squared error (RMSE) in comparison to models generated using 2D, 3D, and 2D+3D.

### Modeling Using the Combined 1D, 2D, and MD Descriptors

The models generated using the combination of all the three descriptors—2D, 3D, and MD—had high accuracy (ANN 78.57%; RF 92.85%) values, balanced accuracies (ANN 87.50%; RF 91.50%), and AUC (ANN 0.91; RF 0.87) values. The compounds predicted as active by both the models included 50267423, 50215590, and 50215720. The MD descriptors alone and in combination with 2D and 3D descriptors performed better in terms of generalization performance.

We also calculated the accuracy of ANN/RF model vis-a-vis the accuracy due to the different input. The accuracy obtained using different input dataset was higher in comparison to the ANN/RF model accuracies, indicating that the ML models generated in the present study would be able to predict outcomes for new unseen data.

### Molecular Dynamics Simulation Analysis of the Most Active Compound

**Figure 3:**
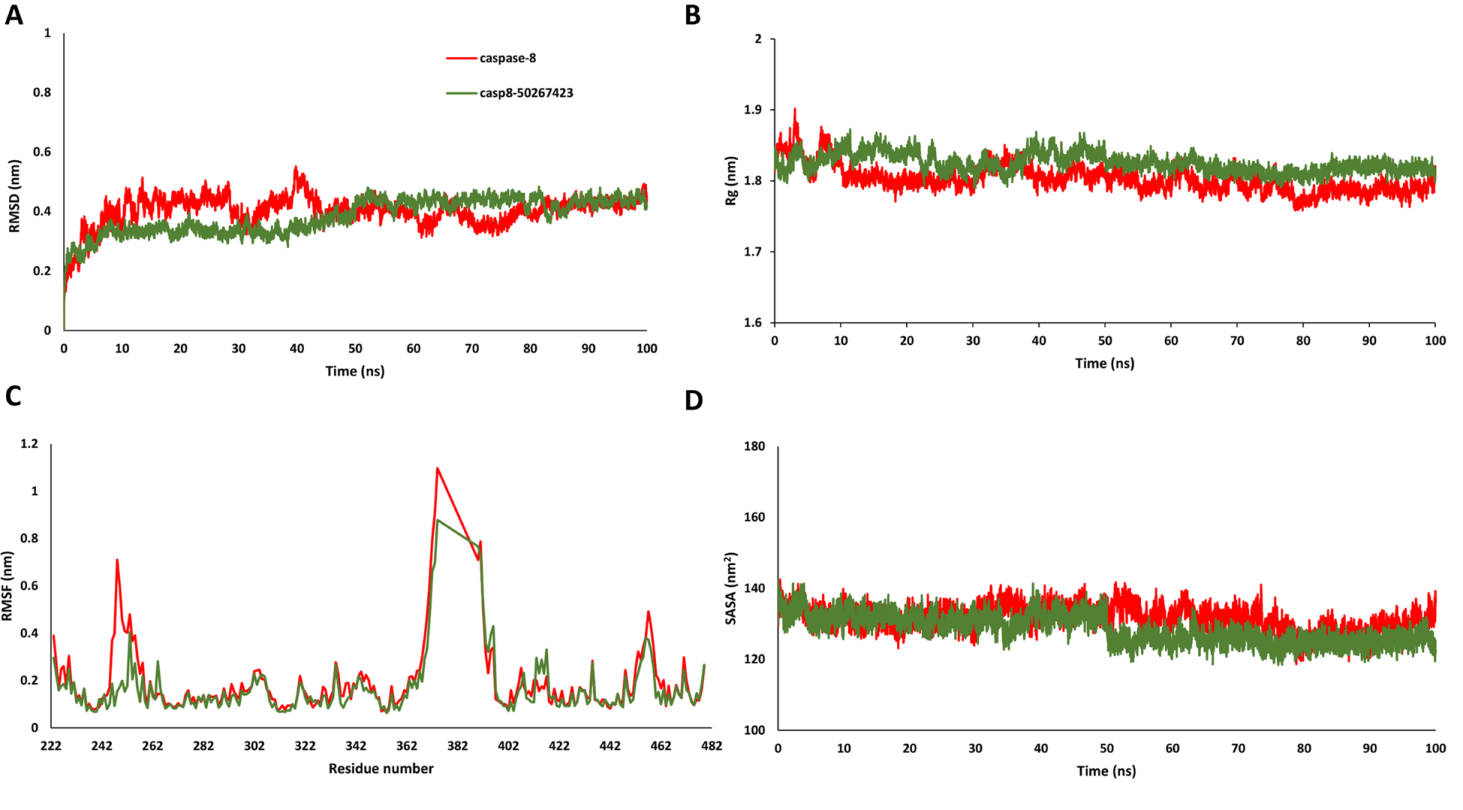
**(A)** Root mean square deviation, **(B)** radius of gyration, **(C)** root mean square fluctuation, and **(D)** solvent accessible surface area plots for caspase8-50267423 complex.

**Figure 4:**
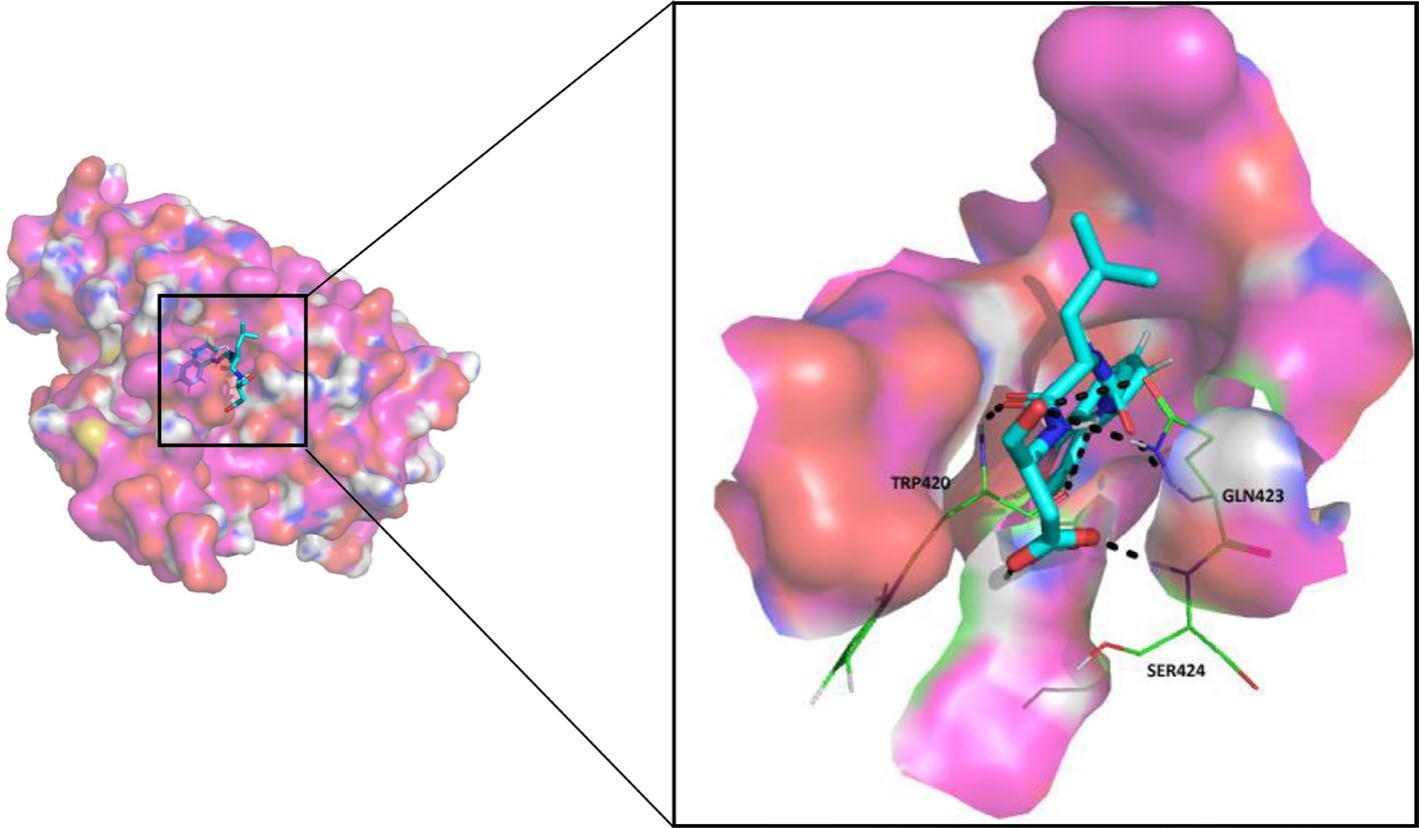
The hydrogen bonding in caspase8-50267423 complex.

**Figure 5:**
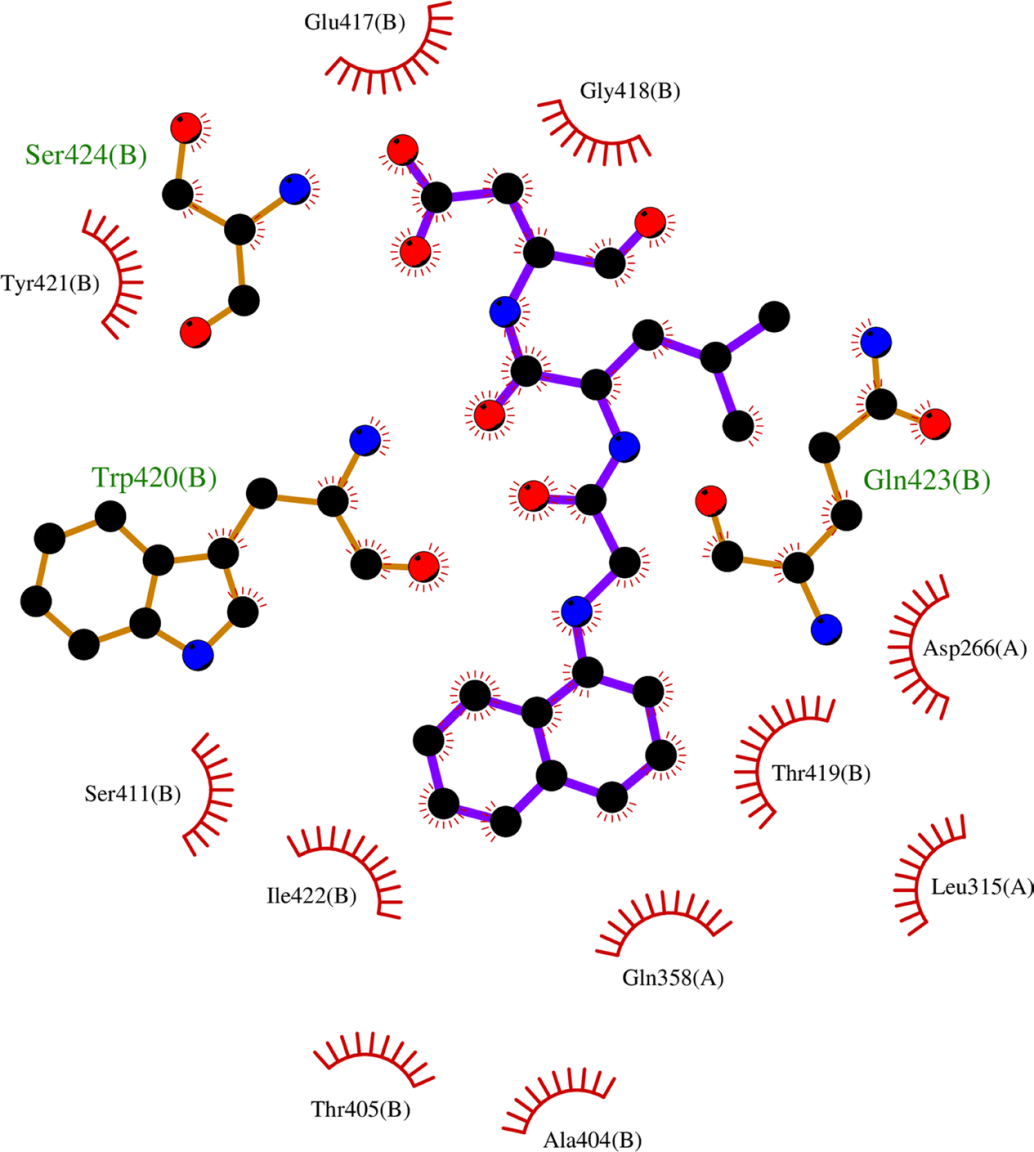
The hydrophobic interactions in caspase8-50267423 complex.

Since most of the ANN and RF models were able to accurately predict this compound 50267423 as active among all the other predicted active compounds, the same was chosen for carrying out long MD simulations. The compound, 50267423, having a docking score of −9.10 kcal/mol was subjected to a 100ns MD simulation for an in depth study of its structural characteristics. As apparent from **Figure 3A**, the unbound caspase-8 protein was unstable, but became stable upon binding with compound 50267423. In both cases, the simulation reached convergence between 10–30ns with RMSD around 0.45 and 0.35 nm for the unbound caspase-8 and caspase-8_50267423 complex, respectively. Next, Rg was calculated to demonstrate the impact of compound 50267423 on the compactness of the protein. The protein had a compact packing in both unbound and bound forms (**Figure 3B**). Root mean square fluctuation (RMSF) analysis was performed to study the fluctuation on residues in the presence of the ligand. **Figure 3C** illustrates the RMSF in free caspase-8 and caspase-8_50267423 complex. The residues had enormous fluctuations in unbound caspase-8 while RMSF values were reasonably low, and the protein was very much stable in the presence of the 50267423 compound. Further, SASA was calculated, which was higher in the case of unbound caspase-8 protein in comparison to the SASA in the ligand-bound protein (**Figure 3D**). Thus, it is evident from the aforementioned results that the caspase-8 protein was highly stable upon binding with compound 50267423. The hydrogen bonding and hydrophobic interaction analyses were carried out for the caspase8-50267423 complex. The ligand formed five hydrogen bonds, which included two bonds with Trp420, two hydrogen bonds with Gln423, and one bond with Ser424, as demonstrated in **Figure 4**. The residues having hydrophobic interactions included Asp266, Leu315, Gln358, Ala404, Thr405, Ser411, Glu417, Gly418, Thr419, Tyr421, and Ile422 (**Figure 5**). The residues having hydrophobic interactions Gln358 and Ser411 have been shown to line the binding pocket in caspase-8 whereas the aromatic group of Ty420 which in the present study is forming two hydrogen bonds with the inhibitor has been shown to help to form the part of the pocket (Watt et al., 1999).

### Identification of Common Pharmacophore and Virtual Screening

**Figure 6:**
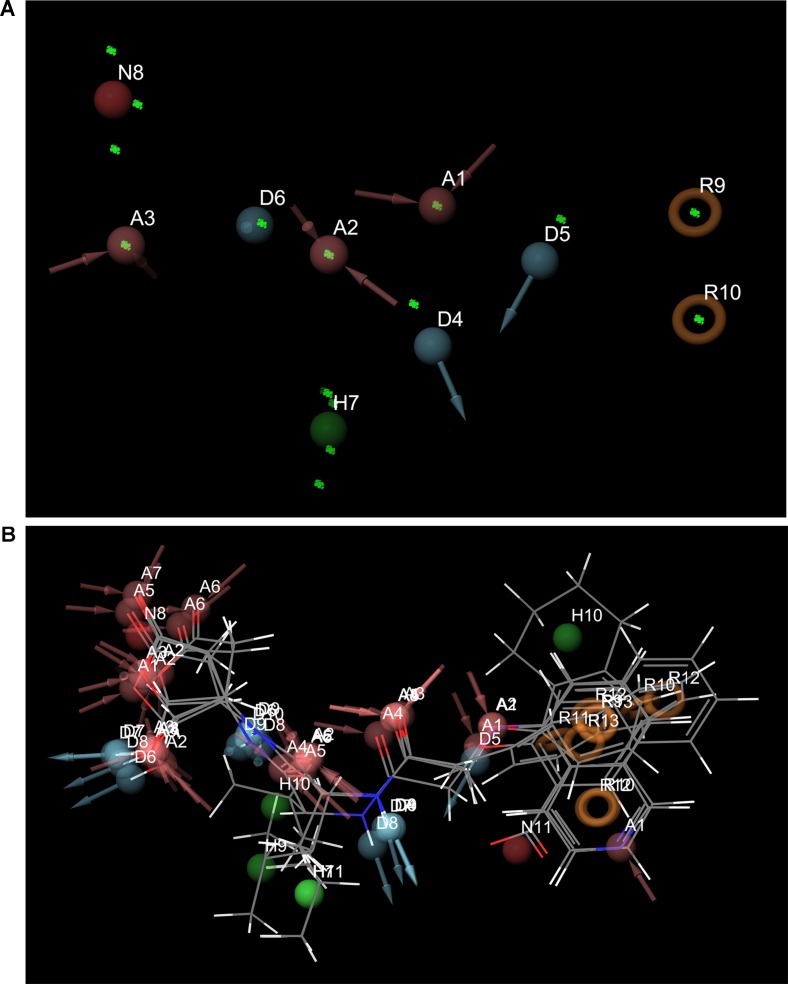
**(A)** and **(B)** The pharmacophoric features of the most active ligand, 50267423, and alignment of other active ligands to the pharmacophore model. The color classification of the features is hydrogen bond acceptor (red), hydrogen bond donor (blue), hydrophobic (green), and aromatic ring (orange).

Pharmacophore search using PharmaGist provided us a high-scoring pharmacophore containing compound corresponding to IDs 50267423 (most active compound) and other active compounds, 50215632, 50215590, 50215720, and 50215896. The pharmacophore model had a total of nine features which included one aromatic ring, one hydrophobic group, two hydrogen-bond donors, and three hydrogen-bond acceptors. This model will be of substantial help in design and development of novel caspase inhibitors. **Figures 6A, B** shows the pharmacophoric features of the most active ligand, 50267423, and alignment of other active ligands to the pharmacophore model. A total of 129 hits were obtained which matched the pharmacophoric features of the most active compound 50267423. The ZINC IDs of the 129 hits have been provided in supporting information. The molecular docking analysis of the top five leads revealed that the XP scores of the compounds ranged between −10.775 and −9.423 (**Table 5**).

**Table 5:** The molecular docking analysis of the top five ZINC compounds obtained after virtual screening using pharmacophore.

ZINC database ID	Glide XP score	Interacting residues (hydrogen bond)
ZINC38200481	−10.775	Arg260 (2), Gln358 (1), Arg413 (3)
ZINC01576107	−10.775	Arg260 (2), Gln358 (1), Arg413 (3)
ZINC02384806	−10.729	Arg260 (1), Gln358 (1), Arg413 (2)
ZINC38570006	−9.702	Arg260 (2), Gln358 (1), Ser411(1), Arg413 (4)
ZINC38569951	−9.423	Arg260 (2), Gln358 (1), Ser411(1), Arg413 (4)

## Discussion

AD is a chronic progressive long-term neurodegenerative disorder that affects millions of people worldwide and thus needs immediate attention. The current drugs available in the market can only temporarily improve upon the symptoms and delay the progression of the disease but could not stop it from progressing and deteriorating the cognitive functions further. This study is based on the hypothesis that incorporating protein-ligand interactions for lead prioritization could lead to identification of compounds with highest binding affinities. In our previous studies, we had used molecular descriptors of chemical compounds to generate ML models for the classification of biologically active compounds (Jamal et al., 2015; Jamal et al., 2017). The properties extracted from MD trajectories have not been yet used for the classification of active compounds. The present work involved generation of ML models based on MD trajectories for prioritization of chemical compounds and lead optimization. Using Glide, we performed molecular docking of caspase-8-associated compounds and performed 10-ns MD simulations of top scoring conformation of each ligand and caspase-8 protein-ligand complex. Several 2D and 3D descriptors were generated, and MD descriptors were obtained from MD simulation trajectories. Various feature selection, Remove Useless filter, CFS, and relief-based attribute selection techniques were used to identify a subset of features having high contribution toward classification. The predictive models were generated using 2D, 3D, and MD descriptors and their combinations, 2D+3D, 2D+MD, 3D+MD, and 2D+3D+MD. Two ML algorithms, ANN and RF, were used for model building. The results obtained indicated that the MD descriptors performed better than 2D and 3D descriptors individually as well as in combinations. The MD descriptors clearly improved the classification performance of the models thus suggesting that the longer simulations as well as the MD descriptors in combination with 2D and 3D descriptors could lead to accurate and efficient lead optimization and prioritization. Another study conducted by Ash and Fourches in 2017 also confirmed the hypothesis that the descriptors extracted from MD trajectories are highly informative descriptors and could be effectively used not only for screening chemical libraries but for drug candidate design and prioritization (Ash and Fourches, 2017). Additionally, we also used a nine-point pharmacophore model consisting of three hydrogen-bond acceptor, two hydrogen-bond donors, one hydrophobic group, and one aromatic ring. This pharmacophore model was used for virtual screening of ZINC library of chemical compounds which led to the identification of 129 hits. The five lead compounds were subjected to molecular docking analysis which resulted in compounds having docking scores between −10.775 and −9.423 indicating that these compounds could be used as potential caspase-8 inhibitors. 

## Contribution to the Field Statement

Dementia is a syndrome, usually chronic or progressive in nature, which leads to decline in cognitive function resulting in loss of ability of thinking and performing routine activities and majorly effects elderly population. Alzheimer’s is a progressive disease during which the symptoms of dementia get worse over time. The current treatment regimen can only improve upon the systems for short term causing a temporary relief though cannot stop the disease from progression. Thus, there is a need of better treatment options which can stop the development of the disease. The high throughput screening studies have resulted in large number of compounds among which many compounds are in clinical trials and can be potential drugs against AD. However, selection of compounds with huge potential activity against Alzheimer’s remains a problem to be addressed. The present study involves generation of predictive classification models using molecular dynamics descriptors which could lead to the identification of bioactive compounds and aid lead optimization and prioritization.

## Supporting Information

Supporting information includes final data set including the molecule identifiers, SMILES and pIC50 values and the training and testing files and the models generated in the present study.

## Data Availability

The raw data supporting the conclusions of this manuscript will be made available by the authors, without undue reservation, to any qualified researcher.

## Author Contributions

SJ conceived, designed and performed the experiments. SJ, SG, and AG analyzed the data. SG contributed reagents/materials/analysis tools. All authors contributed to the writing of the manuscript.

## Conflict of Interest Statement

The authors declare that the research was conducted in the absence of any commercial or financial relationships that could be construed as a potential conflict of interest. 
